# Anesthetic management of acute right heart failure in patients with pulmonary embolism undergoing AngioJet pulmonary embolectomy: a case report

**DOI:** 10.3389/fonc.2024.1477922

**Published:** 2024-11-29

**Authors:** Shasha Zhang, Fangeng Meng, Chao Zhou, Yiwen Zhang, Huaqin Liu, Yuanyuan Rong

**Affiliations:** ^1^ Department of Anesthesiology, The Fourth Hospital of Hebei Medical University, Shijiazhuang, Hebei, China; ^2^ Department of General Internal Medicine, Hebei Province Hospital of Chinese Medicine, Shijiazhuang, Hebei, China

**Keywords:** pulmonary embolism, embolectomy, spasm, right-sided heart failure, case report

## Abstract

**Background:**

Early mortality rate in patients with high-risk pulmonary embolism(PE) is extremely high. Prompt and effective reduction of the thrombus load, and restoration of pulmonary circulation may successfully treat such patients. For patients with hemodynamic instability and high-risk acute PE, the guidelines recommend catheter directed therapy (CDT). Such patients are at increased risk during perioperative period and need considerable attention from anesthesiologists. Herein, we describe a case of acute right heart failure in a patient undergoing AngioJet PE.

**Case summary:**

A 59-year-old woman with lung cancer had been prescribed anticoagulant therapy for PE six months ago. She discontinued using the drugs on her own two months ago. One week ago, she developed chest tightness and shortness of breath, leading to the diagnosis of another PE episode. An AngioJet pulmonary embolectomy and inferior vena cava filter implantation were urgently needed under general anesthesia. During surgery, after inserting the AngioJet Solent catheter into the right lower lobe artery, she developed severe hypotension 5 s after thrombolysis with urokinase, with no obvious improvement after administration of pressor drugs; hence, pulmonary vasospasm was considered. The anesthesiologist implemented a series of resuscitation measures such as discontinuing the surgical stimulation; chest compressions; and administering pure oxygen, vasoactive drugs, and adequate anticoagulation to ensure patient safety during the perioperative period and a smooth operation.

**Conclusion:**

Pulmonary artery spasm caused by AngioJet pulmonary artery embolization is a rare complication and may be life-threatening. Low left ventricular output and acute right heart failure may occur due to pulmonary spasm, which requires early identification, inhalation of pure oxygen, circulatory support, anticoagulation, and thrombolysis.

## 1 Introduction

The annual incidence of acute PE is approximately 100–300/100,000, making it the third most common cardiovascular disease with a mortality rate of 20% (1, 2). The prevalent guidelines recommend thrombolytic therapy or surgical treatment for patients with high-risk PE (2). Interventional surgery for PE is one of the most challenging operations for anesthesiologists. Herein, we report a special case of a patient with PE who underwent AngioJet PE under general anesthesia and experienced severe mixed shock–mainly cardiogenic during the procedure. The hemodynamic stability was maintained through a series of rescue measures, such as stopping surgical stimulation, providing chest compressions, and administering pure oxygen, vasoactive drugs, and adequate anticoagulation. The patient returned to the ward safely. The rescue experience of this case highlights the necessity to strengthen the diagnostic awareness and standardize the resuscitation process of pulmonary artery spasms during AngioJet PE.

## 2 Case presentation

### 2.1 Chief complaints

A 59-year-old female was admitted to our hospital due to PE, aggravated by chest tightness and shortness of breath for 1 week. PE was treated using AngioJet and inferior vena cava filter implantation under general anesthesia.

### 2.2 History of present illness

Six months ago, the patient was hospitalized for meningeal metastasis of lung cancer for more than one month, wheezing for three days, and aggravation of wheezing for one day. On examination, she was found to have PE that was treated with alteplase, followed by low-molecular weight heparin for anticoagulation, and continued anticoagulation for four months after discharge. Two months ago, the patient discontinued the anticoagulation therapy on her own, and one week ago, she experienced chest tightness and shortness of breath, with significantly decreased activity endurance, which progressively aggravated.

### 2.3 History of past illness

Thoracoscopic left upper lobe resection was performed for lung cancer three years ago, and targeted drug therapy was provided after operation. Eight months ago, the patient was hospitalized nine times for symptoms associated with meningeal cancer, underwent lumbar puncture, and was administered intrathecal injections of methotrexate several times. The patient had a history of hypertension of more than 10 years, with a normal blood pressure of 140/100 mmHg. She underwent hysteromyomectomy 14 years ago.

### 2.4 Personal and family history

The patient’s father died of lung cancer at the age of 60 years, and her mother died of coronary heart disease at the age of 57 years.

### 2.5 Physical examination upon admission

On physical examination, the patient had a body temperature of *36.5°C*, heart rate of 106 times/min, respiratory rate of 18 times/min, and blood pressure of 109/82 mmHg; her height was *159* cm, weight was *70* kg, and body mass index was 27.7 kg/m^2^. The patient was brought to the ward on a stretcher, with good consciousness and low spirit. The patient was uncooperative during the physical examination, which revealed fine moist rales in both lungs, tachycardia (the fastest was approximately 130 beats/min), and no edema in the lower limbs.

### 2.6 Laboratory examinations

Laboratory test results showed the following values: electrolytes: blood sodium *134 mmol/L (137-147 mmol/L)* and blood potassium *5.4 mmol/L(3.5-5.3 mmol/L)*; liver and kidney functions: glucose *7.75 mmol/L(3.9-6.1 mmol/L)* and creatinine *77 umol/L(41-73 umol/L)*; coagulation tests: B-type natriuretic peptide precursor *9810.00 ng/L(0-125 ng/L)*, prothrombin time *13.0 s(9.4-12.5 s)*, D-dimer *2.256 mg/L(<0.243 mg/L)*, and fibrinogen degradation product *11.81 mg/L(<5.00 mg/L)*. Troponin I and myoglobin levels were within the normal range. Other laboratory tests showed no obvious abnormality.

### 2.7 Imaging examinations

Chest computed tomography (CT) showed PE in both lungs, postoperative changes in the left lung, a little chronic inflammation in the middle lobe of the right lung, and bullae in the upper lobe of the right lung.

**Figure 1:**
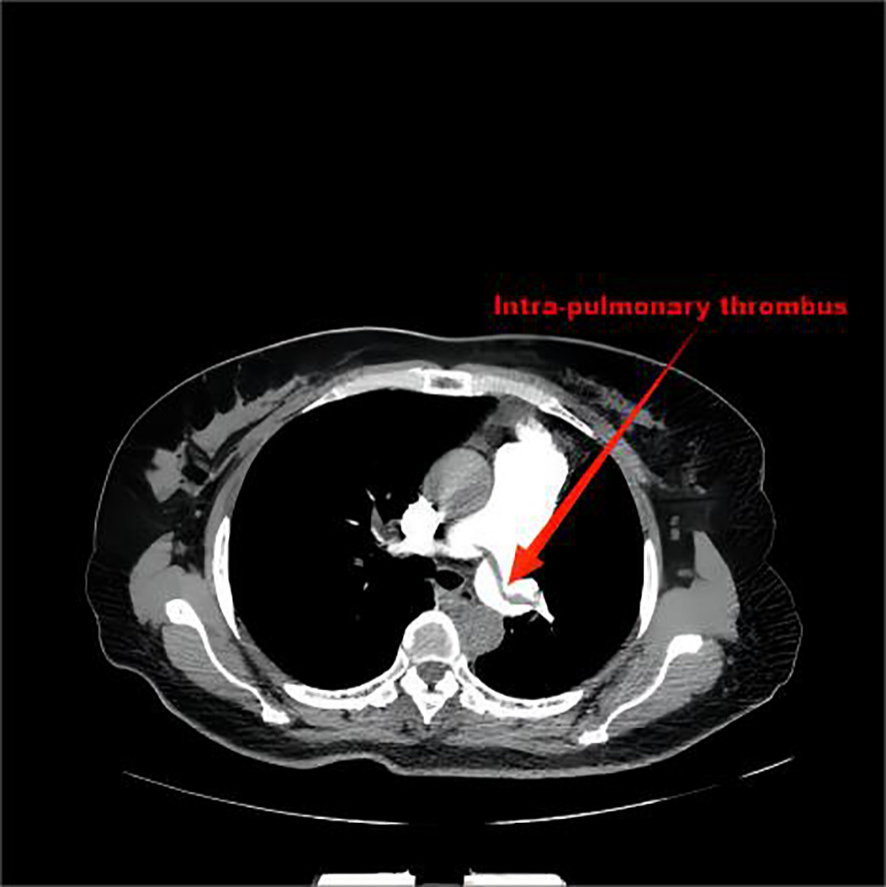
There were strip-filling defects in both pulmonary trunks, which spanned the left and right pulmonary trunks, and filling defects were found in the pulmonary lumens of each lobe of both lungs.

Computer tomography angiography of the pulmonary artery showed that there were strip-filling defects in both pulmonary trunks, which spanned the left and right pulmonary trunks, and filling defects were found in the pulmonary lumens of each lobe of both lungs. The inner diameter of the main pulmonary artery was approximately *37* mm, the width of the left pulmonary artery trunk was approximately *19* mm, and the width of the right pulmonary artery trunk was approximately *21* mm (**Figure 1**).

Color Doppler echocardiography showed that the pulmonary pressure revealed a slight-to-moderate increase, with a small amount of tricuspid regurgitation and an enlarged right heart (–signs of right heart strain). Other related parameters were as follows: transverse diameter of right atrium: *43* mm, anteroposterior diameter of right ventricle: *29* mm, estimated mean pulmonary artery pressure: 51 mmHg, left ventricular ejection fraction: 68%, stroke output: 31 mL/stroke, heart rate: 111 beats/min, and output per minute: 3.5 L/min.

Ultrasonography of lower extremity veins revealed a heterogeneous echo on the inner wall of the right superficial femoral vein, which was suspected to be a wall thrombus and a heterogeneous echo in the right popliteal vein, posterior tibial vein, and calf muscle veins, which were suspected to be due to thrombosis.

Electrocardiogram(ECG) revealed sinus tachycardia (107 beats/min) with right ventricular hypertrophy and nonspecific T wave abnormality. There was a “S1Q3T3” pattern- McGinn-White Sign.

### 2.8 Final diagnosis and treatment

Acute right heart failure caused by pulmonary spasm during PE and embolectomy.

**Figure 2:**
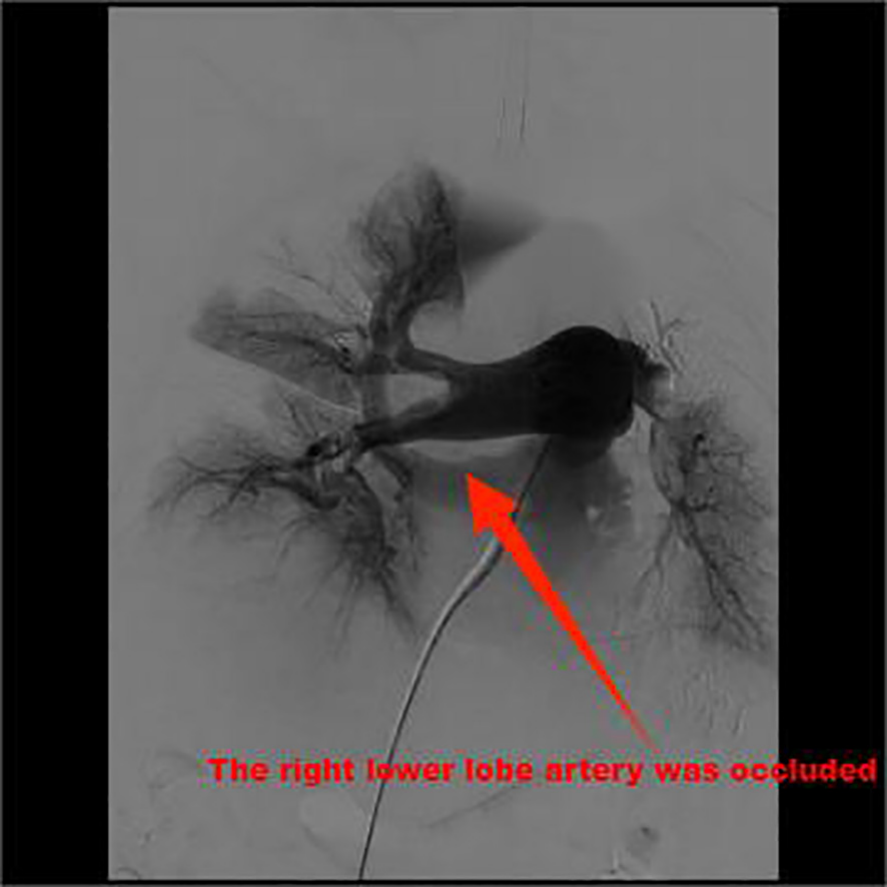
High-pressure angiography revealed that the left pulmonary artery was a banded thrombus and the right lower lobe artery was occluded.

Routine monitoring was initiated as the patient was brought into the operating room at 14:25. The patient’s invasive radial arterial blood pressure was 130/78 mmHg, her heart rate was 101 beats/min, her pulse oximetry was 94% (4 L/min of oxygen inhalation), and arterial blood gas analysis revealed a partial pressure of oxygen (PO_2_) of 109.6 mmHg and partial pressure of carbon dioxide (PCO_2_)of 32.9 mmHg. The patient was administered 0.1 µg/kg/min of norepinephrine before anesthesia induction, and anesthesia induction was initiated at 14:40. The patient was intravenously injected 7 *mg* etomidate, 15 µg sufentanil, and 50 *mg* rocuronium. Following 2 min, a 7.5^#^single-lumen tracheal tube was successfully inserted via the mouth. After successful tracheal intubation, a double-lumen central venous catheter was inserted through the right internal jugular vein. The operation began at 15:20. The left femoral vein was punctured and inserted with a short 8F sheath. The patient was administered 5000 IU of heparin sodium and 10 *mg* of dexamethasone. The left iliofemoral vein and inferior vena cava were unobstructed by hand radiography. A Misgurnus anguillicaudatus guide wire with a single curved catheter was passed through the tricuspid valve to the pulmonary artery trunk, and the pig tail catheter was replaced. High-pressure angiography revealed that the left pulmonary artery had a banded thrombus and the right lower lobe artery was occluded (**Figure 2**). A catheter guide wire was inserted into the right lower lobe artery to replace the supercore guide wire, and an AngioJet solent catheter (Specification Model: SOLENT omni, Boston Scientific Corporation, Two Scimed Place, Maple Grove, Mn USA 55311) was inserted into the inferior lobe artery of the pulmonary artery. Further, 150,000 IU of urokinase was administered for thrombolysis at 16:10. Five seconds later, the patient’s blood pressure dropped to 80/50 mmHg, and her heart rate increased to 123 beats/min. The pump speed of norepinephrine was adjusted to 0.3 µg/kg/min. After administration of 100 mL of compound sodium acetate ringer’s solution, the heart rate increased to 130 beats/min and blood pressure was 83/60 mmHg. Considering cardiac insufficiency, 10 µg/kg/min dopamine was administered following which the heart rate increased to 142 beats/min and blood pressure was 80/60 mmHg. There was no obvious improvement after administering the pressor drugs. A filter was placed in the inferior vena cava, and the sheath was withdrawn. The patient’s blood pressure dropped to 60/30 mmHg, and the percutaneous pulse oxygen saturation dropped to 80%. At 16:35, cardiac compressions were initiated, 1 *mg*/3 min epinephrine was administered, dopamine was stopped, 0.3 µg/kg/min adrenaline was initiated, 1.2 µg/kg/min norepinephrine was pumped, and 190 mL of 5% sodium bicarbonate was administered thrice. The vasoactive drugs adrenaline (0.3 µg/kg/min) and norepinephrine (1.2 µg/kg/min) were administered with blood pressure at 152/100 mmHg, heart rate 149 beats/min, and arterial oxygen partial pressure at 206 mmHg. At 17:45, the patient was returned to the ward with tracheal intubation. The total anesthesia time was *180* min, operation time was *70* min, and rescue time was *62* min (including cardiopulmonary resuscitation for *37* min); 600 mL crystalloid solution was infused, and urine volume was 0 mL. Arterial blood gas analysis was performed intermittently during the perioperative period (**Table 1**).

**Table 1:** Blood gas analysis results of patients with PE after AngioJet pulmonary embolism aspiration.

Time	Before entering the room 12:47	Before anesthesia 16:07	Intraoperative 1 17:00	Intraoperative 2 17:46	Operation day 19:24	The first day after operation 02:37	The first day after operation 19:11	The second day after operation 06:17
pH	7.439	7.199	7.119	7.099	7.060	7.421	7.436	7.503
Potassium (*mmol*/L)	4.65	4.73	4.61	4.96	4.52	3.79	3.99	4.96
Glucose (*mmol*/L)	6.1	12.4	19.8	–	27.8	19.1	15.4	12.7
Lactic acid (*mmol*/L)	0.7	1.4	7.7	8.8	11.7	8.4	2.1	2.4
Hct	35	33.7	37.7	33.8	36	33	32	31
PaO_2_ (mmHg)	109.5	231.5	77.2	170.5	246.6	88.1	132.6	76.8
FiO_2_	0.29	0.7	0.9	0.9	1.0	0.60	0.60	0.33

FiO_2_, Fraction of inspired oxygen; Hct, Hematocrit; PaO_2_, Partial pressure of oxygen.

## 3 Outcome and follow-up

After the patient returned to the ward, the ECG revealed sinus tachycardia (147 beats/min) with a short PR interval, right axis deviation, right ventricular hypertrophy, and abnormal T wave. Blood gas analysis showed that the internal environment was seriously disordered: pH 7.060, PCO_2_ 63.1 mmHg, PO_2_ 246.6 mmHg, lactate 11.7 *mm*ol/L, and erythrocyte pressure volume 0.36. We controlled the circulation stability, strengthened the anticoagulation, and monitored the capacity load and internal environment. The patient was unable to sleep at night and was sedated. On the first day after operation, the vital signs were relatively stable. After discontinuation of sedatives, the patient was awake and breathing spontaneously. The tracheal tube was removed, and oxygen inhalation was delivered with an atomizing mask. On the second day after operation, the patient was conscious and did not complain of discomfort. The following values were noted. Blood gas analysis: pH 7.503, PO_2_ 76.8 mmHg, lactate 2.4 *mmol*/L, and erythrocyte pressure volume 0.31; physical examination: body temperature: *38.8°C*, heart rate: 111 beats/min, breathing: 23 beats/min, and blood pressure: 133/82 mmHg. The patient’s family requested for discharge; at discharge, the patient was prescribed low molecular weight heparin (LMWH) (6000 IU) once every 12 h.

The patient recovered well after discharge. Six months after operation, the patient reported to the neurology department of our hospital for lumbar puncture and intrathecal injection because of meningeal carcinomatosis. At present, she regularly takes anticoagulant drugs and performs daily activities without complaints or discomfort such as chest tightness and shortness of breath.

## 4 Discussion

The early PE-related mortality rate for high-risk patients with PE exceeds 15% (3, 4). Prompt and effective reduction of the thrombus load and restoration of pulmonary circulation is the key to successful treatment of such patients. For patients with PE with contraindication or failure of thrombolysis and hemodynamic deterioration after anticoagulation, CDT should be performed for acute PE. The application of CDT in patients with acute PE is increasing, and simultaneously, the clinical application and research interest of this method are increasing in various countries. CDT includes catheter-directed clot fragmentation, mechanical embolectomy, local thrombolysis, and drug-mechanical combined therapy, which is mainly used to relieve proximal PE, restore pulmonary blood flow, and improve right heart function.

### 4.1 Personalized therapeutic strategy

This study reports a case of PE in which a patient with meningeal metastasis of lung cancer discontinued anticoagulants without consulting the prescriber. Her pulmonary embolism severity index score is 149, with a grade of 5, indicating extremely high risk, with a 30-day mortality risk of 10-24.5%, hence, the patient was diagnosed with high-risk PE.

The patient’s PE treatment plan was tailored to her specific clinical needs. Due to her meningeal metastasis potentially compromising the blood-brain barrier, she faced a significantly increased risk of intracranial hemorrhage (up to 10-20%), making systemic thrombolysis inappropriate. After weighing the risks of thrombosis and bleeding, the Multidisciplinary Team (MDT) decided on a treatment approach involving pigtail catheter mechanical fragmentation, local thrombolysis, and AngioJet PE removal. This approach effectively and swiftly cleared the fresh clots within the pulmonary arteries, restored hemodynamic stability, and reduced the risk of bleeding. Since patients with malignancies are often in a hypercoagulable state and have a high incidence of thrombotic events, studies indicate that the risk of PE in cancer patients can be up to four times higher than in the general population (5). For such patients, long-term anticoagulation with LMWH is recommended (6). The suggested LMWH dose is 100 IU/kg every 12 hours (6). LMWH provides consistent anticoagulation without the need for frequent monitoring, making it particularly suitable for patients who may have difficulty adhering to complex medication regimens. Consequently, for this patient, the chosen long-term anticoagulation regimen was a subcutaneous injection of 6000 IU of LMWH every 12 hours.

### 4.2 Immediate management

The AngioJet Thrombectomy System is a pharmacomechanical peripheral thrombectomy device with active aspiration and Power Pulse™ lytic delivery designed to treat the widest range of thrombosed vessels, rapidly restoring blood flow. Its mechanism of action is as follows. The AngioJet console energizes the pump which sends pressurized saline to the catheter tip; Saline jets travel backwards to create a low pressure zone causing a vacuum effect; Thrombus is drawn into the in-flow windows and the jets push the thrombus back down the catheter; Thrombus is evacuated from the body and into the collection bag. The AngioJet Thrombectomy System is used to break apart and remove thrombus from peripheral arteries and veins ≥ 3.0*mm* in diameter. The Thrombectomy Set has not been evaluated for treatment of PE. It poses a challenge for anesthesiologists. The patient had circulatory instability or even circulatory failure, which was complicated with conditions such as pulmonary hypertension, hypoxemia, pulmonary lobectomy, meningeal cancer. Considering her high-risk PE status and underlying malignancy, the MDT chose AngioJet thrombectomy combined with local thrombolysis. This approach allowed targeted treatment of the thrombus, effectively reducing exposure to systemic thrombolytics and minimizing the risk of serious bleeding complications.

Prompt and accurate identification of pulmonary artery spasm and effective intervention are crucial first steps in resuscitation (**Table 2**). The occurrence of pulmonary artery spasm is closely related to mechanical stimulation from catheters, pressure fluctuations, and endothelial reactions triggered by thrombus disruption. The surgical procedures such as pig tail catheter placement, high-pressure syringe angiography, AngioJet solent catheter embolectomy, and urokinase thrombolysis may induce pulmonary artery spasm in patients (7, 8). The mechanical procedure, sudden aggravation of right heart failure, and stabilization of the circulation after symptomatic treatment support the diagnosis of acute right heart failure caused by pulmonary artery spasm in this patient. At that time, the patient’s vital signs were unstable, and the situation was critical. Considering the urgency of the situation, symptomatic treatment was initiated to rescue the patient, and pulmonary angiography, which could have clarified the cause of circulatory and respiratory failure in this patient, was not performed. Upon suspecting pulmonary artery spasm, surgical procedures should be immediately halted to prevent further mechanical stimulation. Additionally, low-dose inhaled nitric oxide (NO) and administration of prostaglandins can be considered to alleviate the spasm and reduce pulmonary hypertension.

**Table 2:** Pulmonary artery spasm: signs, diagnosis, and management during AngioJet procedures.

Category	Details
Warning Signs	Sudden onset of chest pain
	Dyspnea (difficulty breathing)
	Hemodynamic instability (e.g., hypotension)
	Decreased oxygen saturation
	ECG changes, such as ST-segment elevation or T-wave inversion
	Clinical presentation of respiratory distress and chest discomfort
Diagnostic Criteria	Imaging studies (e.g., angiography) confirming narrowed pulmonary artery
	Hemodynamic monitoring showing sudden increase in pulmonary artery pressure
	Blood gas analysis indicating hypoxemia
	Ruling out other causes of respiratory distress (e.g., myocardial infarction, PE)
Management Steps	Stop AngioJet device immediately to reduce mechanical irritation
	Administer oxygen and ensure adequate ventilation
	Administer analgesics as needed to relieve chest pain
	Consider using nitroglycerin or calcium channel blockers to alleviate arterial spasm
	Use hemodynamic support (e.g., vasopressors) if necessary to stabilize blood pressure
	Provide cautious fluid administration, as fluids may worsen circulatory status
	If spasm persists, consider inhaling low-concentration NO or administering prostaglandins to relieve pulmonary artery spasm
	Perform chest compressions if necessary to assist circulation and reduce thrombus burden through mechanical fragmentation
	Use mechanical extracorporeal support such as veno-arterial ECMO
	If symptoms remain unresolved, consult a cardiologist or interventional radiologist for additional interventions

Understanding the pathophysiological mechanisms behind circulatory failure and implementing prompt supportive treatments are crucial in resuscitation. Pulmonary artery spasm can lead to increased pulmonary vascular resistance and elevated right ventricular afterload, triggering acute right heart failure. Acute dilation of the right ventricle causes the interventricular septum to shift leftward, compressing the left ventricle, restricting its filling, and reducing cardiac output. Simultaneously, a reduction in pulmonary circulation volume, a rapid decrease in left ventricular preload, and diminished cardiac output result in refractory hypotension. Hypoxemia and acidosis further exacerbate pulmonary hypertension, creating a vicious cycle that worsens right heart function, aggravates hypotension, and leads to inadequate tissue perfusion. Insufficient cardiac output caused by acute right heart failure is the primary cause of death in patients with acute PE. During the resuscitation of this patient, active chest compressions, correct vasoactive drugs, and adequate anticoagulation and thrombolysis were the key to successful resuscitation (**Table 3**). The circulatory support treatment plan is as follows: (1) Careful Fluid Resuscitation. Clinical evidence shows that active volume expansion is not only useless, but may worsen the right heart function due to excessive mechanical stretching or reflex mechanism inhibiting myocardial contractility (9); therefore, the symptoms of right heart failure are aggravated after fluid replacement treatment. (2) Choice of Vasopressor Agents: At this time, supportive treatment with vasoactive drugs is extremely important. Norepinephrine can improve the right ventricular function through direct positive inotropic effect and simultaneously improve the systemic blood pressure by stimulating peripheral vascular α receptor and the right ventricular coronary perfusion. Epinephrine has the advantages of both norepinephrine and dobutamine, but has no vasodilating effect of systemic circulation, which may be beneficial for patients with acute PE and shock. Dobutamine and dopamine are beneficial for patients with acute PE with low cardiac index and normal blood pressure; however, caution must be exercised as they can lead to further redistribution of blood flow from blocked blood vessels to unblocked blood vessels, thus aggravating the imbalance of ventilation/blood flow ratio. (3) The Role and Potential Risks of Chest Compressions: Chest compressions not only assist cardiac ejection during cardiopulmonary resuscitation, but also break large thrombi in pulmonary arteries into small thrombi due to the associated mechanical stimulation, thus relieving the thrombus load of pulmonary artery trunk. Need to acknowledge that the detachment of smaller thrombi into the systemic circulation poses a potential risk of stroke in the future. In future cases, adjunctive anticoagulant therapy and thromboprophylactic techniques, such as pulmonary artery filters, may help capture embolic fragments, reducing the risk of small thrombi entering the systemic circulation and thus lowering the incidence of complications like stroke. (4) Extracorporeal membrane oxygenation (ECMO) as a Bridge to Recovery: ECMO is helpful for high-risk PE to maintain the circulation and oxygenation of important organs during acute right heart failure and cardiogenic shock. ECMO support combined with anticoagulation therapy can be used as a bridge to surgery or percutaneous pulmonary embolectomy. An expert group agreed that the best treatment choice of ECMO for PE patients should be individualized according to the multidisciplinary approach (10).

**Table 3:** Case report schedule.

Phase	Time line
Preoperative period	1 Thoracoscopic left upper lobe resection was performed for lung cancer three years ago.
	2 Lumbar puncture was performed and intrathecal injections of methotrexate were administered for meningeal cancer 8 months ago.
	3 Chest CT showed pulmonary embolism in both lungs and postoperative changes in left lung.
	4 Color Doppler echocardiography showed slight-to-moderate increase in lung pressure and an enlarged right heart.
Perioperative period	5 Invasive blood pressure was monitored and arterial blood gas analysis was performed.
	6 Pump norepinephrine before anesthesia induction.
	7 General anesthesia induced by tracheal intubation.
	8 Blood pressure dropped suddenly, and heart rate increased during the operation; vasoactive drugs were administered.
	9 The patient’s circulation deteriorated further; thus, the procedure was discontinued, and she was provided interventions, such as epinephrine pump injection, heparin anticoagulation, and sodium bicarbonate acid correction.
	10 The patient’s blood pressure could not be maintained, and she was administered epinephrine intermittently and chest compressions were provided continuously.
	11 Blood pressure stabilized, vasoactive drug dosage was adjusted, and the patient was returned to the intensive care unit.
Postoperative period	12 Circulation stability was controlled, anticoagulation was strengthened, and attention was paid to volume load and correct internal environment.
	13 On the first day after operation, the patient was awake and spontaneously breathed well, and the tracheal tube was removed.
	14 On the second day after operation, she was conscious, did not complain of discomfort, and was discharged.
	15 The patient was followed-up at 6 months and at 20 months after operation.

### 4.3 Long-term management

In cancer-associated PE patients, long-term anticoagulation is crucial for preventing thrombus recurrence. For patients with incurable malignancies, lifelong anticoagulation may be necessary. In cancer-associated PE cases with a high risk of bleeding, LMWH is preferred over direct oral anticoagulants (DOACs) and vitamin K antagonists (VKAs) due to its superior efficacy in reducing thrombus recurrence and minimizing the risk of major bleeding (11). The recommended dosage is 100 IU/kg administered every 12 hours, with a duration ranging from six months to lifelong treatment (12). To address the patient’s history of discontinuing anticoagulation therapy, additional measures were implemented to enhance her adherence. Comprehensive education for both the patient and her family was emphasized, providing detailed explanations about the importance of anticoagulation therapy and the risks associated with inconsistent medication use. A personalized dosing schedule, tailored to her daily routine (administering low-molecular-weight heparin subcutaneously at 8 AM and 8 PM), was developed. All abbreviations used in this study are listed in **Table 4** for reference. This was supplemented by family reminders or phone alarms. Furthermore, weekly follow-up calls by the medical team were conducted to monitor compliance and offer encouragement. This approach aimed to establish a support system centered on patient self-discipline, family involvement, and consistent medical guidance.

**Table 4:** List of abbreviations used in the manuscript.

Abbreviation	Full Term
PE	Pulmonary Embolism
CDT	Catheter Directed Therapy
CT	Computed Tomography
ECG	Electrocardiogram
PCO_2_	Partial Pressure of Carbon Dioxide
PO_2_	Partial Pressure of Oxygen
LMWH	Low Molecular Weight Heparin
DOACs	Direct Oral Anticoagulants
MDT	Multidisciplinary Team
VKAs	Vitamin K Antagonists
ECMO	Extracorporeal Membrane Oxygenation
NO	Nitric Oxide
TEE	Transesophageal Echocardiography

To prevent further thrombotic events, the MDT recommended placing an inferior vena cava filter during the procedure. After surgery, personalized follow-up is essential, focusing on monitoring anticoagulation adherence, treatment tolerance, and regular imaging to detect any recurrence or progression of thrombus. Follow-up should occur every three to six months, including imaging studies such as echocardiography or CT pulmonary angiography to assess residual thrombi and right heart function. The anticoagulation regimen should be adjusted as needed, based on the patient’s bleeding risk and treatment tolerance, ensuring a careful balance between preventing thrombosis and minimizing bleeding complications.

### 4.4 Areas of improvement and future directions

In this case, the lack of echocardiographic monitoring during the rescue was a notable limitation. Real-time echocardiography is essential during CDT to monitor right heart function and pulmonary artery pressure. However, several practical challenges limit its use in such emergency settings. Many anesthesia departments don’t have dedicated echocardiography equipment, and not all anesthesiologists are trained in its use. In emergencies, such as acute heart failure, time constraints and limited staff can make it difficult to conduct timely cardiac ultrasound assessments. Additionally, transthoracic echocardiography isn’t feasible during chest compressions, and transesophageal echocardiography (TEE) can increase the risk of gastrointestinal bleeding, especially when patients are on anticoagulation with heparin. To overcome these hurdles, close coordination between anesthesia and ultrasound teams is crucial. Designating staff trained in echocardiography for such cases can help speed up assessments. Pre-inserting the TEE probe before giving heparin allows for intermittent monitoring without increasing bleeding risks. If an emergency arises, the designated team member can quickly use the pre-positioned TEE probe for cardiac evaluation, preventing delays. Portable or handheld ultrasound devices, suited for constrained spaces in intervention rooms, provide a practical solution for rapid deployment and monitoring. Regular ultrasound training for anesthesiologists and clear protocols for use in specific scenarios can also help ensure high-quality diagnostic imaging when time is critical.

In this case, NO was not available in the interventional suite, so it was not used to relieve pulmonary artery spasm. NO can be beneficial in such situations as it helps to reduce pulmonary artery pressure and stabilize hemodynamics. For facilities equipped with NO in their operating rooms, its use is recommended as part of the management for pulmonary artery spasm, especially in high-risk patients.

## 5 Conclusion

During the interventional treatment of PE, low left ventricular output and acute right heart failure may occur due to pulmonary spasm, which requires early identification, inhalation of pure oxygen, circulatory support, anticoagulation, and thrombolysis. It is suggested that physicians managing patients with PE should be mindful of pulmonary artery spasm when performing AngioJet pulmonary embolectomy.

## Data Availability

The original contributions presented in the study are included in the article/supplementary material. Further inquiries can be directed to the corresponding author.
